# EMS3D-KITTI: Synthetic 3D dataset in KITTI format with a fair distribution of Emergency Medical Services vehicles for autodrive AI model training

**DOI:** 10.1016/j.dib.2024.111221

**Published:** 2024-12-11

**Authors:** Chandra Jaiswal, Sally Acquaah, Christopher Nenebi, Issa AlHmoud, AKM Kamrul Islam, Balakrishna Gokaraju

**Affiliations:** North Carolina Agricultural and Technical State University, 1601 E Market St, Greensboro, NC 27411, United States

**Keywords:** CARLA simulator, Autonomous vehicles, Point cloud, Machine learning, Deep learning, Traffic scenarios

## Abstract

Contemporary research in 3D object detection for autonomous driving primarily focuses on identifying standard entities like vehicles and pedestrians. However, the need for large, precisely labelled datasets limits the detection of specialized and less common objects, such as Emergency Medical Service (EMS) and law enforcement vehicles. To address this, we leveraged the Car Learning to Act (CARLA) simulator to generate and fairly distribute rare EMS vehicles, automatically labelling these objects in 3D point cloud data. This enriched dataset, organized in the KITTI 3D object detection benchmark format by the Karlsruhe Institute of Technology and the Toyota Technological Institute, improves its utility for training and evaluating autonomous vehicle systems*.*

To bridge the gap between simulated and real-world scenarios, our methodology integrates a wide range of scenarios simulation in CARLA, including variations in weather conditions, human presence, and different environmental settings. This approach enhances the realism and robustness of the dataset, making it more applicable to practical autonomous driving scenarios. The data provided in this article offers a valuable resource for researchers, industry professionals, and stakeholders interested in advancing autonomous vehicle technologies and improving emergency vehicle detection. Furthermore, this dataset contributes to broader efforts in road safety and the development of AI systems capable of handling specialized vehicle identification in real-world applications.

Specifications Table

**Table:** 

Subject	Computer Vision and Pattern Recognition
Specific subject area	3D Object Detection in Autonomous Vehicle for the Emergency Vehicles such as Ambulance, Police car, with non-EMS objects such as Pedestrian, Car and Bikers
Type of data	Image 1382px *512 px, Point Cloud data in NumPy Binary, Label and Calibration information with .txt
Data collection	The CARLA simulation was employed to generate various scenarios involving EMS vehicles alongside other traffic entities, ensuring a balanced presence of EMS vehicles within the traffic environment. Diverse traffic situations were emulated to capture objects within each scene. Four ego vehicles were deployed at different locations on the map to introduce randomization. These vehicles were equipped with both cameras and LiDAR sensors and were programmed to follow predefined paths across the entire map of a specific town to ensure comprehensive coverage. All other traffic objects and their movements were controlled by CARLA's AI, allowing for fully randomized behaviors to simulate realistic traffic conditions. The ego vehicles captured a total of 3,000 images, saving every third frame to introduce variation between captured scenes. Raw data collected through this method was then converted into the KITTI 3D format, facilitating its immediate use in AI model training. This conversion ensures compatibility with existing deep learning frameworks, which natively support the KITTI format, thereby minimizing the need for additional data preprocessing efforts.
Data source location	North Carolina A&T State University Greensboro, North Carolina, USA*.*
Data accessibility	Repository name: Zenodo Data identification number: 10.5281/zenodo.13824217 Direct URL to data: https://zenodo.org/records/13824218 The dataset is licensed under the Creative Commons Attribution 4.0 International (CC BY 4.0) license. This license allows for the redistribution and reuse of the dataset, provided proper credit is given to the original creators.
Related research article	C. Jaiswal, H. Penumatcha, S. Varma, I. W. AlHmoud, A. K. Islam and B. Gokaraju, “Enriching 3D Object Detection in Autonomous Driving for Emergency Scenarios: Leveraging Point Cloud Data with CARLA Simulator for Automated Annotation of Rare 3D Objects,” SoutheastCon 2024, Atlanta, GA, USA, 2024, pp. 1137-1143*,* doi: 10.1109/SoutheastCon52093.2024.10500173 [*1*].

## 1 Value of the Data

• This dataset addresses a significant gap in most publicly available computer vision datasets by overcoming the challenge of limited data for rare objects, specifically focusing on emergency vehicles such as ambulances and police cars.
• This dataset is designed for seamless integration into deep learning model training workflows, with a specific focus on identifying emergency vehicles such as ambulances and police cars. It has been preprocessed and formatted into the widely used KITTI 3D format, ensuring compatibility with existing AI frameworks. As a result, researchers and developers can utilize the dataset directly without requiring extensive data preparation, massaging, or preprocessing. This streamlined approach significantly reduces the time and effort needed to prepare the dataset for training, allowing for a more efficient and straightforward application in EMS vehicle detection tasks.
• The synthetic dataset offers a wide variety of real-world scenarios, including diverse traffic conditions, weather variations, and a range of geographic landscapes, all generated using CARLA's 8 towns. This setup allows researchers to utilize different combinations of training, testing, and validation datasets from these towns, which are provided separately. By selecting various combinations, researchers can tailor their model training to meet specific requirements, resulting in more robust and well-trained models suited to different environments and challenges.
• This dataset has the potential to enhance the safe passage of EMS vehicles and improve overall road safety, which is a critical concern for the autonomous driving industry. By enabling more accurate detection and response to emergency vehicles, the dataset contributes to the development of AI models that can prioritize EMS vehicles, ensuring quicker and safer navigation through traffic. This advancement is crucial for creating autonomous systems that can effectively respond to emergency scenarios, ultimately strengthening road safety in real-world applications.

## 2 Background

Object detection in point cloud data is a critical component of autonomous driving systems, enabling accurate identification and localization of objects in 3D space. LiDAR, commonly used for capturing point clouds, allows self-driving cars to perceive their surroundings and detect vehicles, pedestrians, cyclists, and other obstacles in real time, ensuring safe navigation and collision avoidance.

Existing datasets, such as KITTI [2], Waymo [3], and nuScenes [4], primarily focus on a few common object categories, such as vehicles, pedestrians, and bicycles [5,[6], [7], [8]]. However, many less frequent but important objects, like emergency vehicles or strollers, are underrepresented [1]. Detecting these rare objects is essential for the overall safety and performance of autonomous vehicles, as missing them can lead to delayed reactions and accidents. Addressing this gap in object detection is crucial for enhancing the real-world applicability of autonomous driving systems. This challenge motivated the creation of our dataset, which offers better representation of EMS vehicles rare objects. By addressing the lack of these critical classes in existing datasets, our dataset aims to improve object detection capabilities for autonomous driving systems, ensuring more accurate identification and response to emergency vehicles and enhancing overall road safety. A smaller subset of a similar dataset from CARLA Town 12 was initially used to demonstrate the performance of the deep learning model and address the research gaps highlighted in the paper published at the IEEE Southeast Conference [1]. Following the publication, we expanded the work to include all publicly available CARLA towns, resulting in an extended version of the dataset for public use.

## 3 Data Description

CARLA is an open-source simulator for autonomous driving research, offering realistic urban environments, diverse scenarios, and sensor simulations. Developed by the CVC, it enables testing and validation of self-driving algorithms, making it a key tool for advancing autonomous vehicle technology [9]*.* CARLA provides a total of 12 towns, with Town08 and Town09 not available for public use. Town11 and Town12 are very large maps designed for more complex scenarios. Table 1 shows a description of the eight towns we used for recording our data. Each town has its own folder.

**Table 1:** The CARLA Towns.

Town	Specifications and characteristics of the town
1	A small, simple town with a river and several bridges.
2	A small simple town with a mixture of residential and commercial buildings.
3	A larger, urban map with a roundabout and large junctions.
4	A small town embedded in the mountains with a special “figure of 8” infinite highway.
5	Squared-grid town with cross junctions and a bridge. It has multiple lanes per direction. Useful to perform lane changes.
6	Long many lane highways with many highway entrances and exits. It also has a Michigan left.
7	A rural environment with narrow roads, corn, barns and hardly any traffic lights.
10	A downtown urban environment with skyscrapers, residential buildings and an ocean promenade.

Each folder for these town contains two subfolders after navigating to “vehicle.tesla.model3.master” → “kitti_object”

**Fig. 1:**
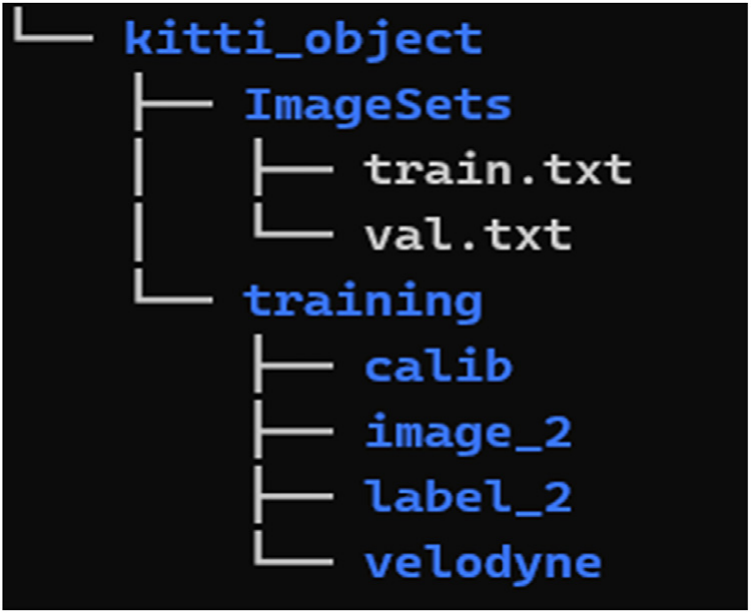
Folder structure.

**Fig. 2:**
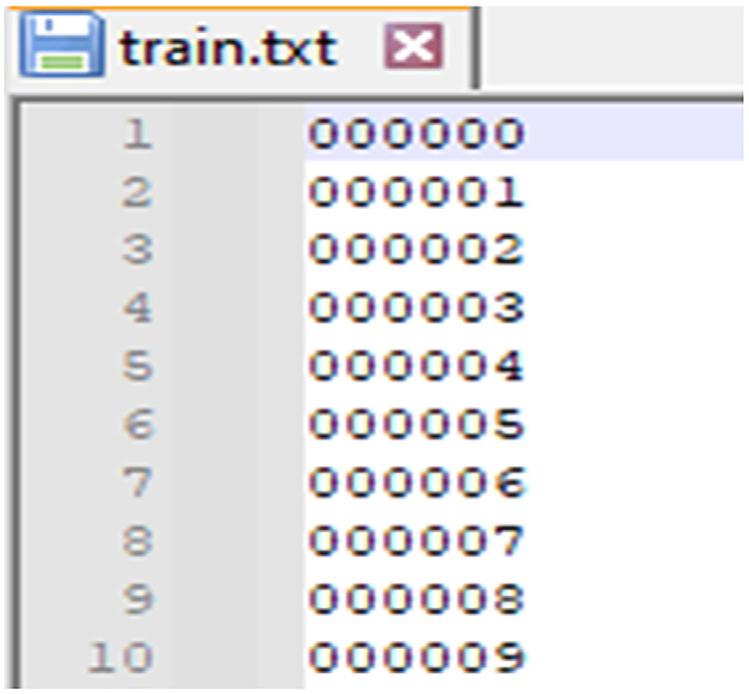
Training frame list.

1. ImageSet:
2. training:

Folder Structure of the dataset is shown in Fig. 1:

1. ImageSets folder contains text files that define the splits of the dataset into training, validation, and testing sets. Each file lists the indices of the data samples (by file ID) to be used for specific tasks. The contents often include:

**1.1 train.txt:** A list of sample IDs to be used for training. This has all the Sample IDs so you can change them based on your need for training (Fig. 2).

**Fig. 3:**
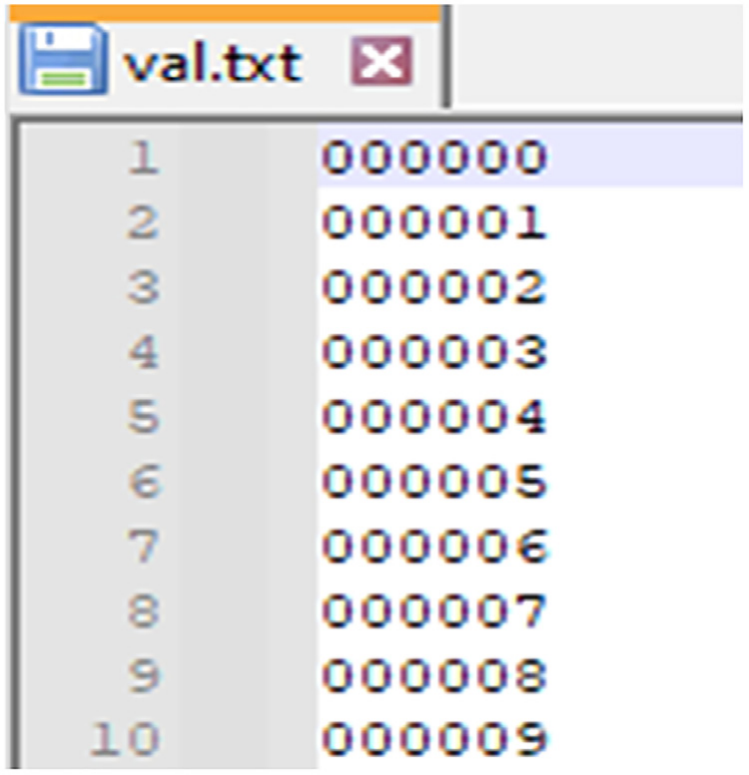
Validation frame list.

**1.2 val.txt:** A list of sample IDs to be used for validation. This has all the Sample IDs so you can change them based on your need for training (Fig. 3).

2. training folder contains the actual data required for training the model. Below are the subfolders and their content

**Fig. 4:**
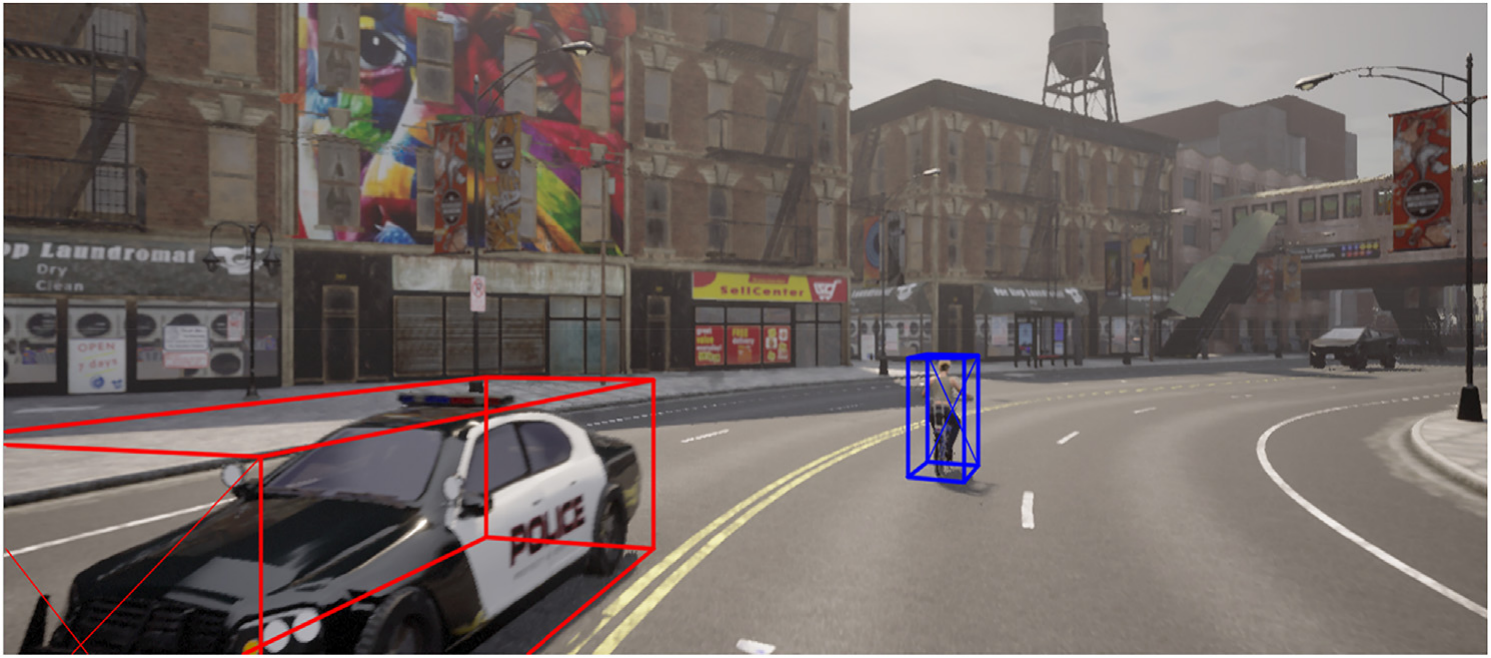
Sample Image with bounding boxes – Police car and pedestrian.

**Fig. 5:**
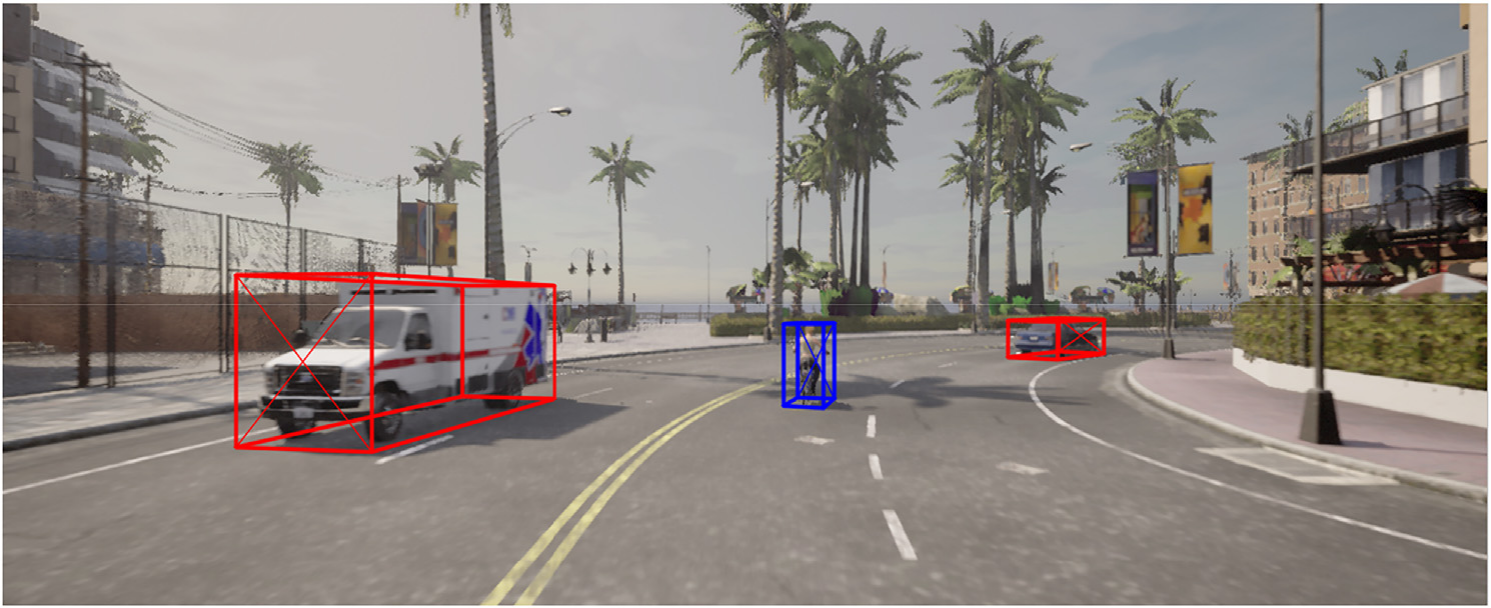
Sample Image with bounding – EMS vehicle, car, and pedestrian.

**2.1 image_2:** Contains RGB images from the front camera of the vehicle. The images are used for 2D and 3D object detection tasks. Files are named by their ID (i.e., 000001.png). Figs. 4 and 5 shows samples of the captured RGB images along with bounding boxes of 3D objects.

**Fig. 6:**
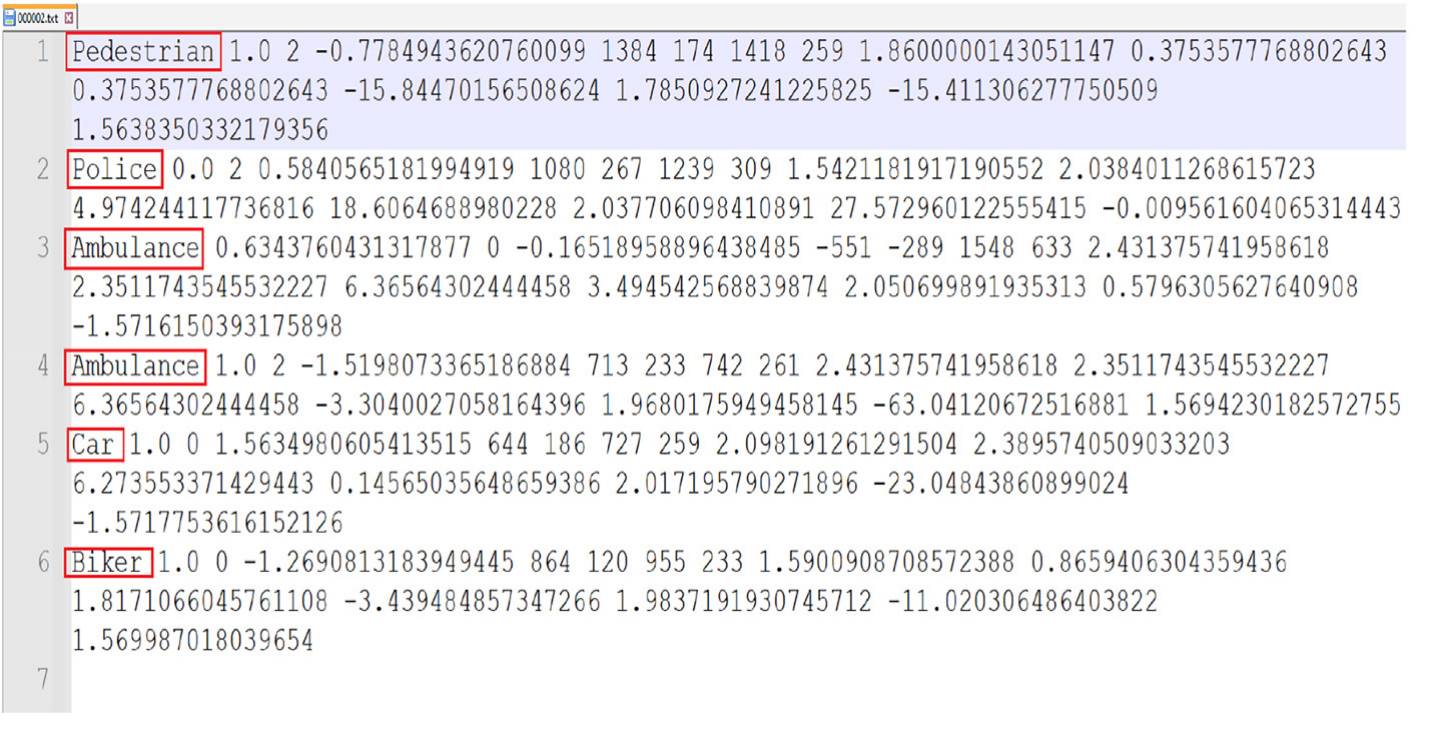
Label for the frame.

**2.2 label_2:** Contains ground truth labels for 2D and 3D object detection. Each label file corresponds to an image and includes information like object type (car, pedestrian, etc.), bounding box coordinates, object dimensions, and location in 3D space. Files are named using the same ID as the corresponding image (i.e., 000001.txt). Fig. 6 shows the ground truth label attributes of each frame.

Below is a breakdown of each of these attributes:

**2.2.1 type:**

The type of object (e.g., Car, Pedestrian, Cyclist, etc.). This field tells the model what class the object belongs to.

**2.2.2 truncated:**

A float value between 0 and 1, indicating how much of the object is truncated (i.e., cut off by the image boundaries). A value of 0 means the object is fully visible, and 1 means the object is completely truncated.

**2.2.3 occluded:**

An integer (0, 1, 2, 3) representing the level of occlusion, as detailed below:

• 0: Fully visible
• 1: Partly occluded
• 2: Largely occluded
• 3: Unknown

The criterion for the occlusion is defined below

Let P represent the number of points within the bounding box, and $center_{x}$​ is the x-coordinate of the bounding box center.

$$\mathrm{Occlusion}\left(P,{\mathrm{center}}_{x}\right)\;=\;\left\{ \begin{array}{c} 0,\;P>20\;\\ 1,\;125<P+{\mathrm{center}}_{x}<250\;\\ 2,\;P+{\mathrm{center}}_{x}<125\;\\ 3,\;\mathrm{othe}\mathrm{rwise}\; \end{array}\right.$$

**2.2.4 alpha:**

The observation angle of the object in the image plane, ranging from [-pi, pi]. This angle helps determine the object's orientation relative to the camera.

**2.2.5 bbox (xmin, ymin, xmax, ymax):**

The 2D bounding box of the object in the image, represented by four values: xmin, ymin: Coordinates of the top-left corner of the bounding box. xmax, ymax: Coordinates of the bottom-right corner of the bounding box. This is used for 2D object detection.

**2.2.6 dimensions (** h **,** w **,** l **):**

The 3D dimensions of the object in meters:

h: Height

w: Width

l: Length

**2.2.7 location (** x **,** y **,** z **):**

The 3D location of the object in the camera coordinate system (in meters). These values represent the object's center (typically the bottom-center of the object's bounding box) in 3D space.

**2.2.8 rotation_y:**

The rotation of the object around the Y-axis (yaw) in radians, representing its orientation in 3D space relative to the camera.

**Fig. 7:**
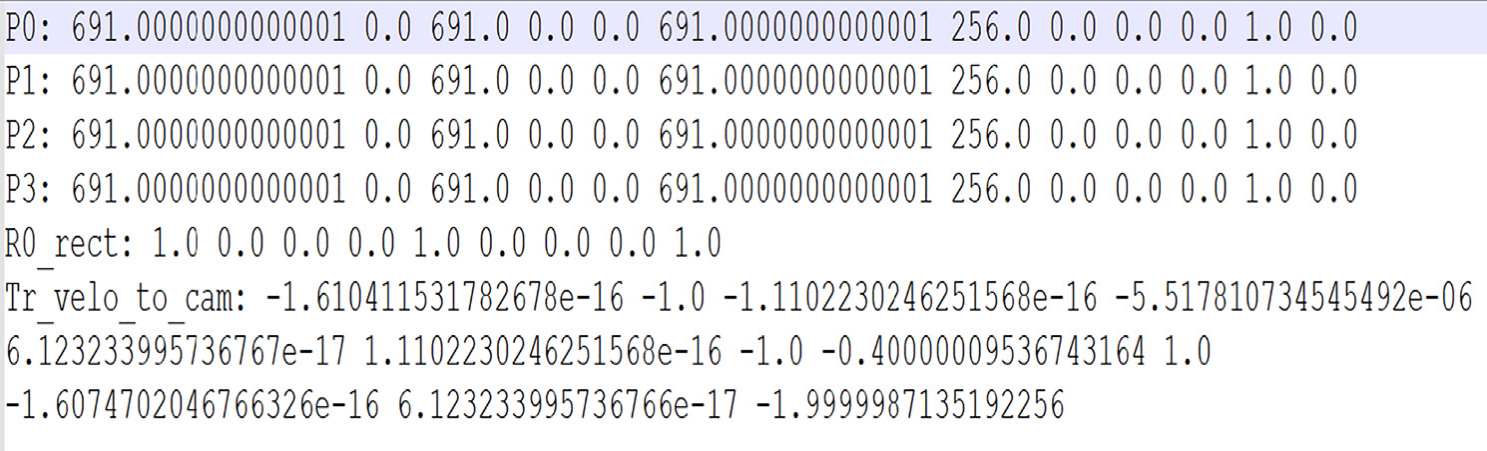
Calibration matrix.

**2.3 calib:** Contains calibration files that map 3D points from LiDAR or 3D space to 2D images. These files are necessary for converting the raw point cloud data into the camera image space. Each file corresponds to an image and provides intrinsic and extrinsic camera parameters. Files are named by their ID (i.e., 000001.txt). Fig. 7 shows the calibration matrix of LiDAR and camera.

**Fig. 8:**
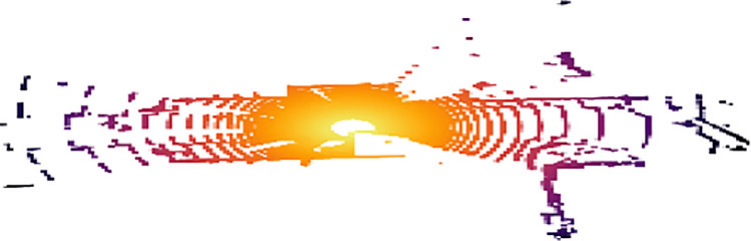
Point cloud visualization using Open3D.

**2.4 velodyne:** Contains the LiDAR point cloud data in binary format (.bin files). Each file contains 3D point cloud data captured by the LiDAR sensor for the corresponding image frame, named by the image ID, such as 000001.bin. Fig. 8 illustrate a visulaization of 3D point cloud using Open3D.

**Fig. 9:**
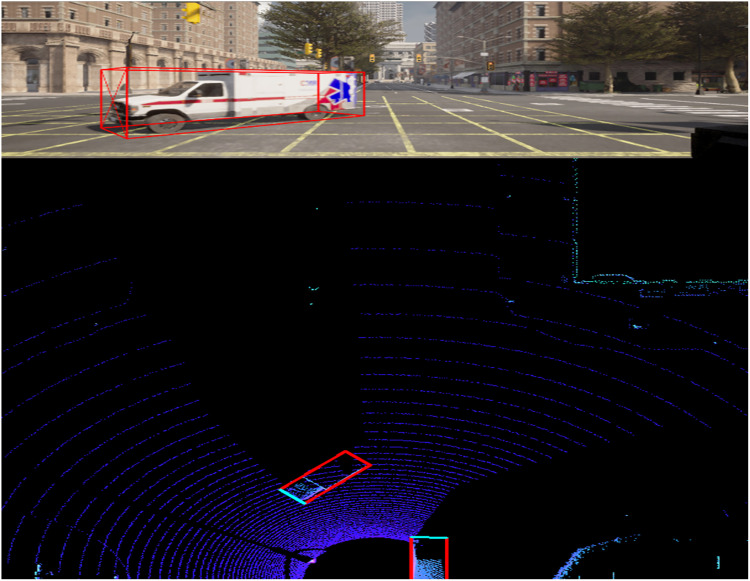
BEV LiDAR and camera using same frame.

**Fig. 10:**
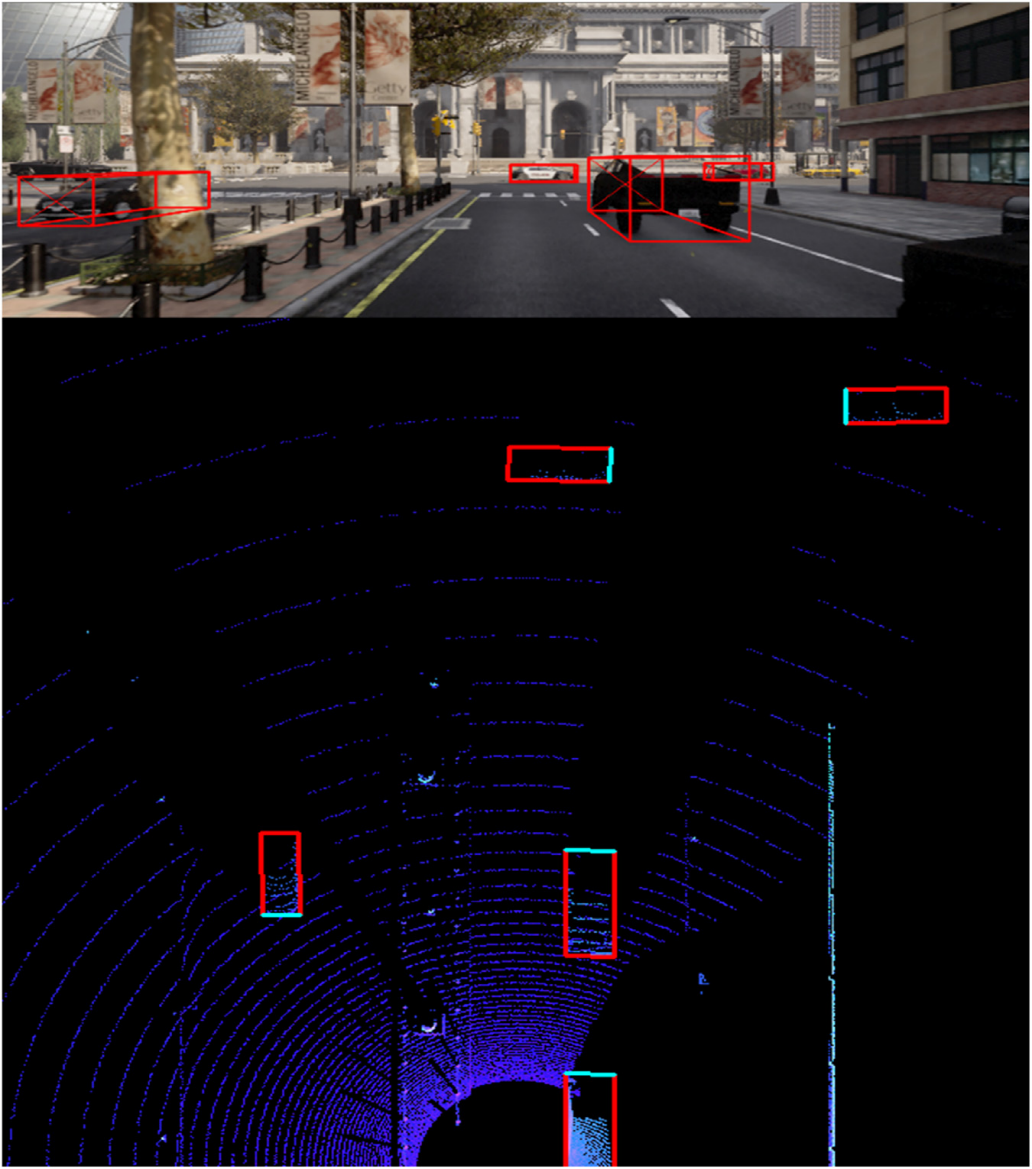
BEV LiDAR and camera using same frame.

A bird eye view (BEV) visualization is shown in Figs. 9 and 10 for the LiDAR and camera using the same frame.

Point cloud data is stored in Binary format of a NumPy version. Below is the format of the Bin NumPy values.

**2.4.1**x, y, z:

The 3D coordinates of the point in the LiDAR coordinate system.

**2.4.2 intensity:**

The reflectance or intensity value of the laser pulse that hit the object at this point. It indicates how strongly the point reflects the laser beam.

## 4 Experimental Design, Materials and Methods

**Fig. 11:**
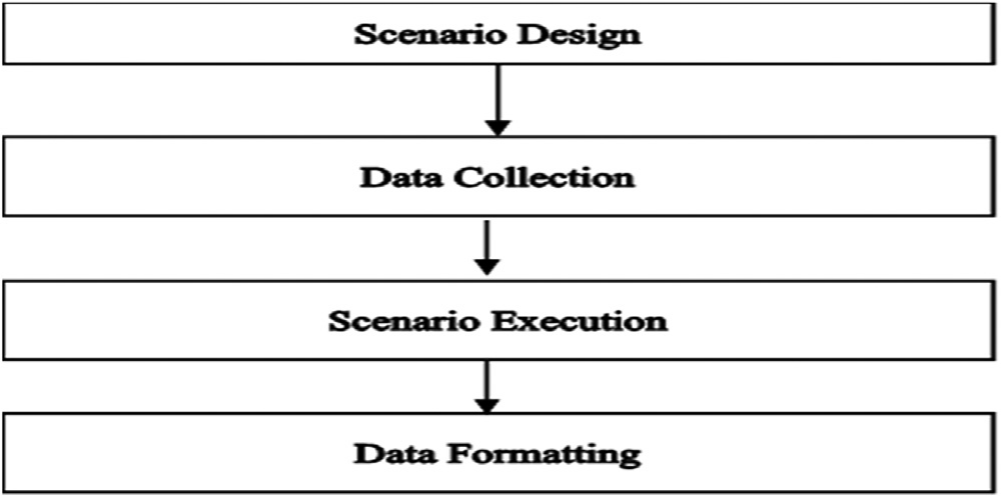
Methodology for data creation.

CARLA was used for running the simulation scenario. Fig. 11 shows the methodologies for creating the synthetic dataset.

**1. Design Rationale**

• **Objective of Dataset Creation**: As highlighted in the background section, EMS vehicles are rarely represented in publicly available datasets, making it challenging to obtain real-world data with sufficient instances of these vehicles due to their infrequent appearance in typical scenarios. To address this gap, we developed a dataset that provides an increased presence of EMS vehicles, specifically designed to support the training of deep learning models in recognizing and understanding EMS-related objects and scenarios effectively.
• **Considerations for Fair Representation**: To achieve a balanced presence of EMS vehicles in the dataset, we implemented a strategy within CARLA that increased the frequency of EMS vehicles in each scenario. Specifically, we configured 25% of the vehicles in each simulated scenario as EMS vehicles. These EMS vehicles were randomly assigned spawn points across the town to ensure they appeared at various locations, providing diverse contexts. This randomized distribution ensured that the EMS vehicles were spread uniformly throughout the environment, enabling the ego vehicle to encounter them frequently and from different angles. Consequently, the data gathered from camera and LiDAR sensors captured EMS vehicles in various scenarios, enhancing the dataset's utility for model training. Below in Table 2, we present the object distribution in the scenes, illustrating that 25% of the objects are from the EMS vehicle category. A total of 80 Objects were spawned in all available eight maps for CARLA. These objects that were in forward position from the ego vehicle were considered for the label generation.

**Table 2:** Distribution of categories.

Group Cat	Total Obj	Class	Initial Pos	Class %	Group %
Vehicle	27	Vehicle	random	33.75	50%
	13	Vehicle	specific	16.25	
EMS Related Object	10	Ambulance	Random	12.5	25%
	10	Police	Random	12.5	
Pedestrian	20	Walker	Random	25	25%
• **Scenario Creation Strategy:** We utilized CARLA's pre-designed towns, each crafted to reflect unique real-world contexts. For instance, Town01 represents a simple town layout featuring a river and several bridges, while Town10 provides a high-definition map of an urban downtown environment with skyscrapers, residential buildings, and an ocean promenade. These diverse town environments simulate various traffic scenarios, including intersections, roundabouts, and large junctions, enabling the dataset to encompass a wide range of real-life situations. This approach enhances the dataset's contextual variety, making it more robust for modeling complex, realistic traffic conditions.

**2. EMS-Related Object Characteristics**

**Fig. 12:**
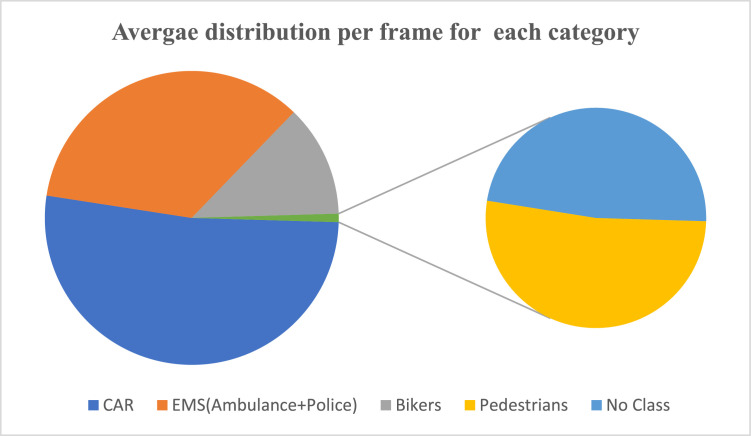
Average distribution per frame for each category.
• **View Angles and Perspectives:** We used the yaw angle from the labels to determine the orientation of the bounding boxes relative to the camera's viewpoint. Fig. 13 illustrates the definition of the yaw angle.

• **Object Density and Frequency:** To ensure diverse object representations and minimize redundant data, we captured every third frame, selecting only distinct frames while reducing unnecessary captures. For each frame, we recorded the camera and LiDAR positions, capturing all scene objects and their bounding boxes. In total, 8,000 frames were collected, with the average object count per frame detailed in Table 3 and Fig. 12. EMS vehicle presence was also measured, averaging close to 2 EMS vehicles per frame.

**Table 3:** Average object/frame.

Category	Total Object	Avg. Object/Frame
CAR	21,747	2.718375
EMS (Ambulance + Police)	14552	1.819
Bikers	5,151	0.643875
Pedestrians	204	0.0255
No Class	188	0.0235

**Fig. 13:**
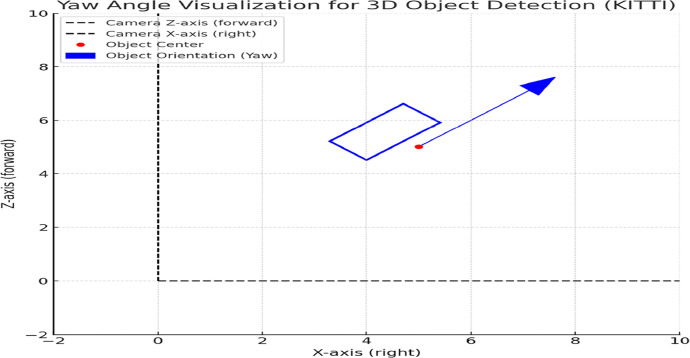
yaw angle visualization.
• The **dashed lines** represent the camera's coordinate system: the Z-axis (forward) and the X-axis (right).
• The **red dot** marks the center of the detected object.
• The **blue arrow** shows the object's orientation based on its yaw angle, indicating the direction the front of the object is facing relative to the camera.
• The **blue rectangle** represents the bounding box of the object, rotated by the yaw angle to align with the object's actual orientation in 3D space.

In this example, the yaw angle is set to 45 degrees (π/4 radians), causing the object to face slightly to the left of the camera's Z-axis.

Using the above definition, we divided the angle for different views of the bounding boxes. Viewing angle direction (θ) can be defined as below

$$\mathrm{Direction}\left(\theta \right)=\;\left\{ \begin{array}{c} \mathrm{Front}\left(F\right),\;-\frac{\pi }{4}\;\leq \;\theta \;\leq \;\frac{\pi }{4}\\ \mathrm{Left}\;\mathrm{Side}\left(\mathrm{LS}\right),\;\frac{\pi }{4}<\;\theta \;\leq \;\frac{3\pi }{4}\\ \mathrm{Right}\;\mathrm{Side}\left(\mathrm{RS}\right),\;\frac{3\pi }{4}\;\leq \;\theta <\;-\frac{\pi }{4}\;\\ \mathrm{Back}\left(B\right),\;\mathrm{othe}\mathrm{rwise} \end{array}\right.$$

The chart below shows the distribution of the viewing angle for the EMS Vehicle (Ambulance + police).

**Fig. 14:**
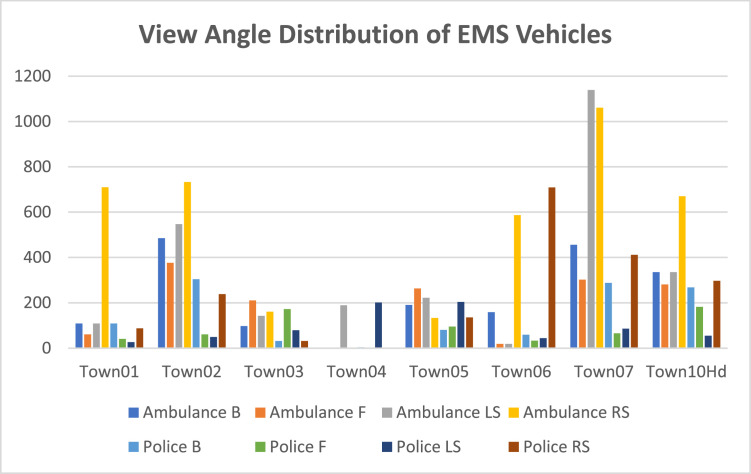
View Angle Statistics for EMS vehicle.

Fig. 14 shows the distribution of the EMS vehicles view angle

**3. Dataset Composition and Statistics**

• **Summary Statistics**: We have collected 41,842 objects, and their distribution is shown in Table 4.

**Table 4:** Object distribution.

Category	Total Object
CAR	21,747
Ambulance	10103
Police	4449
Bikers	5,151
Pedestrians	204
No Class	188
• **Distribution of the objects categories across various Towns:**Fig. 15 shows the spread of the different objects in various Carla Towns.

**Fig. 15:**
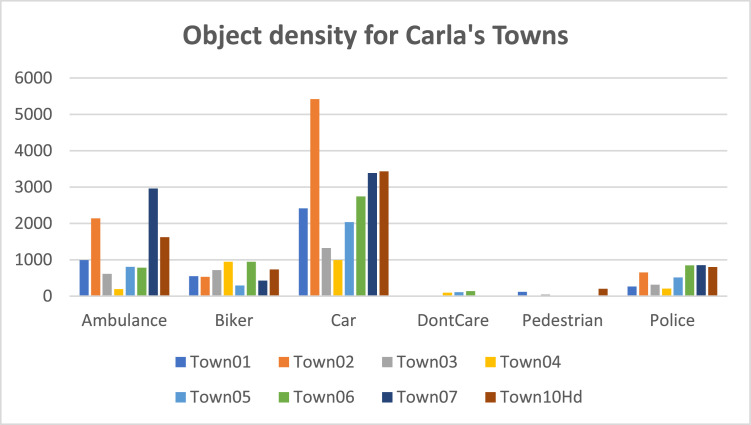
Object density for each CARLA Towns.

**Fig. 16:**
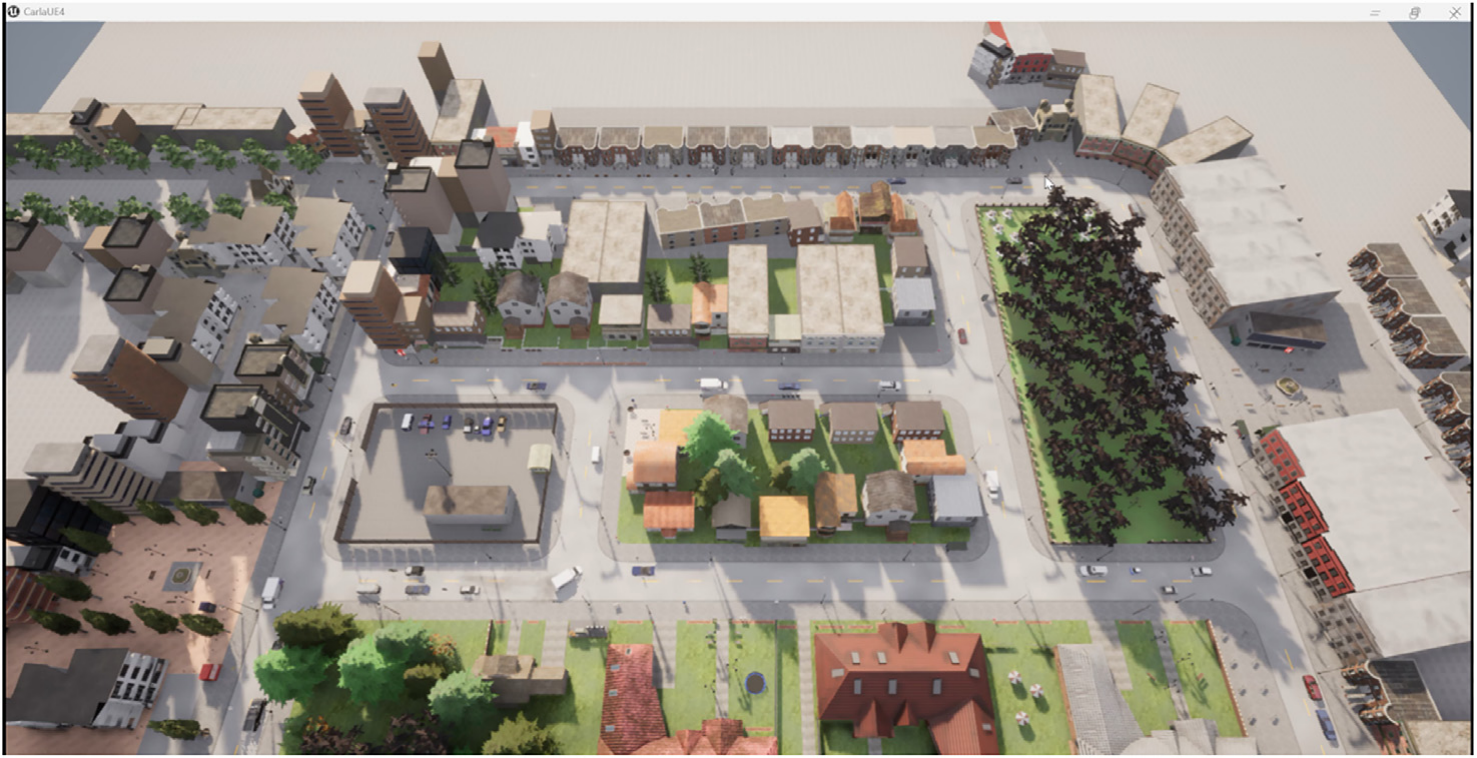
Aerial view for Town03.

Fig. 16 shows Town03 in aerial view during the data recording mode as an example.

## Limitations

This dataset is generated using CARLA simulation and synthetic data and may not fully reflect real-world conditions with 100% accuracy. Additionally, most publicly available datasets do not contain these specific EMS vehicle classes, which further emphasizes the need for careful attention and thorough validation when building models. Various validation techniques will be required to bridge the gap between the synthetic data and real-world scenarios, ensuring the models are robust and reliable for practical applications. This process is essential to ensure the effective use of this dataset in real-world autonomous driving systems.

## Ethics Statement

The authors have adhered to the ethical requirements for publication in Data in Brief. This work does not involve human subjects, animal experiments, or data collected from social media platforms.

## CRediT Author Statement

**Chandra Jaiswal:** Conceptualization, Methodology, Formal Analysis, Writing – Original Draft, **Sally Acquaah:** Writing – Original Draft Writing, **Christopher Nenebi:** Writing – Original Draft **Writing, Issa AlHmoud:** Supervision, Writing – Review & Editing, **AKM Kamrul Islam:** Supervision, **Balakrishna Gokaraju:** Supervision, Funding Acquisition.

## Data Availability

ZenodoEMS3D-KITTI: Synthetic 3D Dataset in KITTI Format with a fair distribution of Emergency Medical Services Vehicles for Autodrive AI Model Training (Original data).
